# A Randomized, Double-Blind, Controlled Trial Assessing If Medium-Chain Triglycerides in Combination with Moderate-Intensity Exercise Increase Muscle Strength in Healthy Middle-Aged and Older Adults

**DOI:** 10.3390/nu15143275

**Published:** 2023-07-24

**Authors:** Keiichi Kojima, Haruna Ishikawa, Shinji Watanabe, Naohisa Nosaka, Tatsushi Mutoh

**Affiliations:** 1 Central Research Laboratory, The Nisshin OilliO Group, Ltd., Yokohama 235-8558, Kanagawa, Japan; h-ishikawa@nisshin-oillio.com (H.I.); shi-watanabe@nisshin-oillio.com (S.W.); n-nosaka@nisshin-oillio.com (N.N.); 2 Department of Aging Research and Geriatric Medicine, Institute of Development, Aging and Cancer, Tohoku University, Sendai 980-8574, Miyagi, Japan; tmutoh@akita-noken.jp; 3 Research Institute for Brain and Blood Vessels, Akita Cerebrospinal and Cardiovascular Center, Akita-City 010-0874, Akita, Japan

**Keywords:** medium-chain fatty acids, medium-chain triglycerides, muscle strength, walking ability, frailty, sarcopenia

## Abstract

An adequate nutritional intake is recommended for the prevention of physical frailty and sarcopenia. In particular, medium-chain fatty acids (MCFAs) are reportedly important for muscle strength in nursing home residents. However, the effects of MCFAs on healthy adults at risk for frailty remain unknown. Hence, a randomized, placebo-controlled study was conducted to investigate the effects of 12 weeks of medium-chain triglycerides (MCTs) intake and walking on muscle mass and function in healthy, sedentary, middle-aged and older adults with a low body mass index. Three MCT intake groups with different amounts of octanoic and decanoic acid intake were compared with a control group. After 12 weeks, knee extension strength increased in all groups, with the increases in all MCT intake groups being significantly higher than those in the control group (*p* < 0.05). Grip strength significantly increased from baseline in the MCT 6 g/day intake group (*p* < 0.05). The combination of aerobic exercise and MCT intake may be effective in preventing decline in muscle strength and promoting increase in muscle strength as they can improve muscle energy production, thereby contributing to the maintenance of good health for middle-aged and older adults at high risk for frailty and sarcopenia.

## 1. Introduction

Frailty, which can occur when elderly persons experience a decline in physical and mental health, is associated with a higher risk of hospitalization and mortality [1]. In Japan, lifespans for both males and females with mild to moderate disability due to the progression of frailty have increased between 1995 and 2004 [2]. A systematic review and meta-analysis revealed that frailty is associated with increased healthcare costs among community-dwelling older adults [3]. Therefore, preventing frailty among the elderly is a desirable outcome in terms of reducing healthcare expenses. The mean score of muscle strength in healthy older adults is reportedly 20–40% lower than that of young adults [4], indicating age-related deterioration of muscle composition and function.

Fried et al. defined frailty as a clinical syndrome in which three or more of the following criteria are present: unintentional weight loss (10 lbs in past year), self-reported exhaustion, weakness (grip strength), slow walking speed, and low physical activity. Xue et al. further associated physical frailty with decreased intake (decreased appetite) and sarcopenia, identifying chronic undernutrition (inadequate intake of protein and energy) as a cause of weight loss, and sarcopenia as a cause of exhaustion, low physical activity, slow walking speed, and weakness (e.g., reduced grip strength) [5]. These relationships are shown in the proposed frailty cycle, in which nutritional intake and frailty prevention are inseparable, as sarcopenia leads to decreased nutritional intake (decreased appetite).

Several reports have observed an association between nutritional status and frailty, with cross-sectional studies showing an association between low energy intake and frailty and an increased risk of frailty with low energy intake [6,7]. Prospective studies have also demonstrated an increased risk of frailty and mortality associated with low energy intake [8]. Japanese clinical guidelines [9] recommend that appropriate nutritional intake, especially protein intake, may be effective in the prevention and suppression of sarcopenia, with high-energy, high-protein foods being recommended for the elderly. However, systematic reviews have shown inconsistent effects of protein supplementation on muscle mass and muscle function [10,11]. In addition, some elderly have difficulty increasing their dietary intakes due to decreased appetite and digestive function [12,13], making it beneficial for them to eat small nutritious meals for easily maintained muscle mass and function.

Medium-chain fatty acids (MCFAs) are straight-chain saturated fatty acids with carbon numbers between 6 and 12 in length. Medium-chain triglycerides (MCTs), which are composed of MCFAs, are oils that are preferentially converted into energy in the body after ingestion, unlike long-chain fatty acids (LCFAs) that are found in regular oils [14]. The characteristics of MCT enables an increase in energy intake, even in elderly individuals with decreased appetite. MCT has been used for the medical nutritional supplementation of premature infants and postoperative patients. Moreover, studies have shown that nursing home residents who consumed MCT had improved grip strength and walking speed [15]. Consumption of MCFAs increases the active form of ghrelin [16], which strongly stimulates the secretion of the growth hormone (GH) promoting skeletal muscle protein synthesis through insulin-like growth factor 1 (IGF-1). MCTs are an effective nutrient for the elderly as they are easy to digest, energy-rich, and help to maintain and enhance muscle strength and function. However, the effects of continued MCT intake in healthy older adults are not yet fully understood. While there are reports of increased plasma acyl ghrelin levels with the administration of 3 g/day of octanoic acid triglyceride [17], the impact of lower doses of MCT on muscle mass and strength remains unclear. Moreover, two MCFAs, octanoic and decanoic acids, reportedly affect metabolism through different mechanisms [18], but no reports have been found on the effects of decanoic acid-dominant MCTs on muscle.

In this study, we tested whether continuous MCT intake is effective in maintaining and preventing loss of skeletal muscle mass and strength in healthy community-dwelling middle-aged and older adults. Along with continuous MCT intake, subjects engaged in moderate-intensity walking, and the impact of different MCT amounts and MCFAs compositions was examined. A combination of walking (an aerobic exercise that is easier to perform than resistance training) and MCT intake can maintain or suppress the reduction in skeletal muscle mass and strength in middle-aged and older adults at risk of frailty. Moreover, foods containing MCFAs as well as an active lifestyle may help middle-aged and older adults maintain their health.

## 2. Materials and Methods

### 2.1. Ethics

This study was reviewed and approved by the Clinical Research Ethics Committee of Yōga Allergy Clinic prior to initiation (IRB number: 21000023, approval date: 13 May 2022). The summary of this study was registered in UMIN-CTR (UMIN000046861). This study was conducted at the Kowa Clinic Vision Center Tamachi by a contract research organization (Huma R&D Co., Ltd.) under the guidance of a physician. The research institute and the contract research organization conducted the study in compliance with the Declaration of Helsinki, the Ethical Guidelines for Medical and Health Research Involving Human Subjects, and the Japanese Act on the Protection of Personal Information.

### 2.2. Subjects

After obtaining the consent of patients to participate in the study, the principal investigator enrolled subjects who met the selection criteria and were able to comply with the management procedures during the trial period.

The selection criteria were Japanese males and females (1) aged between 60 and 75 years at the time of obtaining written consent, (2) with body mass indexes (BMIs) between 19 kg/m^2^ and 24 kg/m^2^, (3) who exercised or walked less than once a week and for less than 30 min at a time, (4) who do not engage in childcare or nursing care on a daily basis (more than 4 h per week), and (5) who were fully informed of the purpose and content of the study, had the capacity to consent, understood the study well, voluntarily volunteered to participate, and agreed to participate in the study in writing.

Exclusion criteria included subjects who (1) had been instructed by a physician to refrain from walking or exercising; (2) were currently receiving medication or inpatient treatment for any serious illness; (3) were currently under the supervision of a physician for exercise or diet therapy; (4) could develop allergies to soybean, shrimp, crab, wheat, milk, egg, almond, squid, orange, cashew nut, kiwifruit, beef, sesame, salmon, mackerel, chicken, banana, pork, peach, yam, apple, or gelatin; (5) had a current or history of drug or alcohol dependence; (6) were currently visiting the hospital for mental disorders (e.g., depression) or sleep disorders (e.g., insomnia, sleep apnea syndrome, and so on), or had a history of mental illness; (7) were current smokers or had smoked in the past 3 months; (8) had irregular lifestyles due to night work or shift work; (9) had an extremely irregular lifestyle in terms of eating, sleeping, and so on; (10) were very fussy eaters; (11) had a weight fluctuation of ±5 kg or more within a 3-month period; (12) had knee surgery or leg problem and used a walking cane daily; (13) had a serious current or previous illness, such as brain disease, malignant tumor, immunological disease, diabetes, liver disease (e.g., hepatitis), renal disease, cardiac disease, thyroid disease, adrenal disease, or other metabolic disease; (14) had used (or was currently using) health foods, supplements, or medicines that affect muscle strength, walking function, or fatigue reduction in the past 3 months; (15) consumed or were currently consuming any health oil (e.g., coconut oil or MCT oil) that may enhance any of the dietary components being evaluated, retroactively, in the past 3 months; (16) had participated in other clinical trials within 3 months prior to the date of consent or have plans to participate in other clinical trials during the study period; (17) had donated more than 200 mL of blood within 1 month or more than 400 mL of blood within 3 months prior to the date of obtaining consent; (18) were currently pregnant or lactating, or may become pregnant or lactate during the study period; (19) had difficulty in complying with recording on various questionnaires; (20) were judged to be unsuitable as subjects based on clinical laboratory values and measurements at the time of screening; or (21) were judged by the investigator to be unsuitable as subjects.

### 2.3. Target Number of Cases

In a previous study, in which 21 elderly nursing home residents with a BMI of 23 kg/m^2^ or less and 65 years of age or older were given 6 g of MCT contained in a test food per day for 3 months, increased grip strength was confirmed [19]. In the current study, the daily intake of MCT was either 6 g or 2 g, which is the same or one-third of the MCT intake in the previous study. The number of patients required for the current study was set at 30 per group, for a total of 120 cases, based on the expectation that the number of cases would increase, and that dropouts and ineligible subjects would occur during the course of the study.

### 2.4. Test Design

Tests were conducted as part of a randomized, double-blind, placebo-controlled, parallel-group study. The study food allocation manager assigned subjects via stratified randomization to three test food intake groups and one control food group. The stratification factors were age, sex, and blood albumin levels. The test food allocation manager kept the study food allocation list strictly confidential until key opening, and blinding was maintained for all parties, except the study food allocation manager.

During the study period, subjects were instructed as follows. (1) Refrain from excessive exercise (except for the exercise intervention described below) and maintain a normal lifestyle. (2) Ensure non-routine household chores that require physical effort (e.g., major cleaning, redecorating/repairing the house, shoveling snow, and so on) are limited to once a week. In addition, when household chores that fell under the above categories are performed, record the details on the daily lifestyle survey form. (3) Limit day outings for recreation to twice a month, and limit consecutive outings involving overnight stays to 3 days or less and once a month. In the event that leisure activities that fall under the above categories are undertaken, or in the event that the number of outings exceeds the stipulated number of days for unavoidable reasons, record the details on the lifestyle survey form. (4) Maintain the usual lifestyle and environment (such as eating, drinking, sleeping, working, and so on, other than the aforementioned test meals prior to participation in the study) as much as possible. (5) Do not take any medicines, supplements, or health foods that may affect muscle mass/muscle strength, walking function, or fatigue reduction. If such a ingestion was unavoidable, contact the consultation desk in advance if possible. If ingested, record the details on the lifestyle survey form. (6) During the study period, record the regular intake of unrestricted medicines, supplements, or health foods on the lifestyle survey form, and continue the habit. If you are not currently taking these additional substances, refrain from intaking them for the study period. However, if this becomes unavoidable, notify the consultation desk in advance if possible. (7) During the study period, do not consume health oils (coconut oil, MCT oil, and so on) that could possibly enhance the dietary components that are to be evaluated. (8) During the study period, dairy products could be consumed as long as the amount and frequency remained as usual. In the unlikely event of a change, record the details on the lifestyle survey form. (9) During the study period, do not to participate in any other clinical trials. (10) Do not donate blood or blood products during the study period. (11) If you wish to receive immunizations, health checkups, or physical examinations during the study period, report this in advance to the consultation desk and record it on the lifestyle survey form.

### 2.5. Test Foods and Dietary Surveys

One package of the test food contained a total of 3 g of long-chain triglyceride (LCT) (Nisshin Canola Oil; The Nisshin OilliO Group, Ltd., Tokyo, Japan) or/and MCT (Nissin MCT Oil, Nissin MCT-C10R; The Nisshin OilliO Group). From the 0-week test day until the day before the 12-week test, two packets of test food were administered daily, comprising one packet after two meals among breakfast, lunch, or dinner, for a total of two packets per day. The subjects in each group were administered one of the following test foods as their experimental food: control food, decanoic acid food, low-dose octanoic acid food, or high-dose octanoic acid food. The amounts of LCT, MCT, and MCFA in MCT per 6 g of fat in the test foods consumed per day are shown in Table 1.

During the 12-week intervention period, dietary surveys were conducted at baseline, and at 4, 8, and 12 weeks after intervention. Surveys were carried out using a simplified self-administered dietary history questionnaire (brief-type self-administered diet history questionnaire: BDHQ) from which nutrient intakes were calculated.

### 2.6. Exercise Intervention

During the examination period, subjects in all groups were instructed to walk outdoors for 40 ± 10 min, 2 days per week, and to record any other activities in a lifestyle survey. The walking speed was to be maintained at the usual pace. Additionally, it was specified that exercise should not be performed on consecutive days whenever possible.

In the event of inclement weather conditions such as heavy rain or extreme heat that would make safe outdoor walking difficult, the following indoor alternative exercises were permitted:

(1) Walking on a treadmill or other walking machines at a normal walking speed for a designated period of time.
(2) Foot stomping on a flat surface for a designated time at a tempo equivalent to normal walking without moving.

If exercise was substituted for (1) or (2) above, or, if exercise could not be carried out due to unavoidable reasons, the reason was recorded on the lifestyle questionnaire.

### 2.7. Life Surveys

During the study period, the subjects were asked to record their daily living conditions, such as consumption of test foods, physical condition, medications, exercise, and alcohol consumption on the lifestyle questionnaire.

### 2.8. Adverse Events

Adverse events were investigated daily during the subjects’ consumption of the test foods by using a lifestyle questionnaire. In addition, laboratory values were also checked for abnormal fluctuations. If an adverse event was observed, the principal investigator took appropriate measures as necessary and categorized the adverse events into three levels: mild—not requiring any intervention and allowing the trial to continue; moderate—requiring some form of intervention; and severe—intolerable, thus necessitating the discontinuation of the trial. Furthermore, adverse events were classified into six levels: none, probably none, possible, probably present, present, and evaluation impossible. Only adverse events determined to have a causal relationship with the test food (excluding those classified as probably none or none) were considered as side effects.

### 2.9. Measurements

During the 12-week intervention period, body composition, grip strength, knee extension strength, and walking ability were measured before intervention, at and 4, 8, and 12 weeks after intervention.

Body composition was measured using a body composition analyzer InBody S10 (InBody Japan Inc., Tokyo, Japan).

Grip strength was measured using the Grip-D Smedley digital grip strength tester TKK-5401 (Takei Scientific Instruments Co., Ltd., Kamo, Japan) in accordance with the Ministry of Education, Culture, Sports, Science and Technology Implementation Guidelines, which recommended the following new physical fitness test.

(1) The subject is asked to be in a standing position, with the upper limb in a drooping position and the forearm in a mid-rotational position.
(2) The grasping bar of the grip strength meter should be adjusted so that it is half the length from the base of the subject’s thumb to the tip of the index finger.
(3) The measurement of maximum grip strength involved two consecutive measurements in the following order: right hand, left hand, 1 min rest, right hand, left hand. The recorded representative value was the average value.

Knee extension strength of both right and left legs was measured using a lower limb muscle strength measuring device (Locomo Scan-II; ALCARE Co., Ltd., Tokyo, Japan).

As an indicator of walking ability [20], the timed up and go test (TUG) was performed using the Multi-Timer T.T.K.5801 device (Takei Scientific Instruments Co., Ltd., Kamo, Japan) as follows.

(1) Have the subject sit deeply in a chair and start walking on the examiner’s cue.
(2) The examiner instructs the subject as follows: “Please walk as quickly as possible, go around the pole located 3 m ahead, in any direction you prefer. Once you return, please sit down on the chair immediately”.
(3) The time from when the subject’s body starts moving until their buttocks made contact with the chair was measured. The measurement was taken once.

### 2.10. Statistical Analysis

The obtained data were analyzed using the methods specified in the statistical analysis plan prepared prior to the start of the study. The nutrient intake levels were presented as mean ± standard deviation for pre-intervention (week 0) and post-intervention (weeks 4, 8, and 12) calculated values. In addition, measurements of body composition, muscle strength, and TUG were taken, calculating the changes from pre-intervention (week 0) as the reference to post-intervention (weeks 4, 8, and 12), and the values were presented as mean ± standard deviation.

Nutritional intake during the intervention period was subjected to multiple comparisons to compare the control and test diet groups. First, the presence or absence of homogeneity of variance was confirmed for each data using Levene’s test, then Steel’s test was conducted if significance was found, and Dunnett’s test was used if it was not significant.

For other measured variables, first, multiple comparisons were conducted among the four groups for the value at week 0 of intake. If no significant differences were found, a linear mixed-effects model was utilized (with fixed effects for diet and time of measurement, and a random intercept for subjects) to examine the interaction between diet effects and time effects for the repeated measurements. If no interaction effect was observed, a linear mixed-effects model equation without the interaction term was used to confirm the presence of diet effects for the repeated measurements (weeks 4, 8, and 12). If diet effects were detected, multiple comparisons were conducted among the four groups at each time point (weeks 4, 8, and 12). If an interaction effect was observed, a one-way analysis of variance (ANOVA) was performed to confirm the diet effects, and if significant differences were found, multiple comparisons among the four groups at each time point (weeks 4, 8, and 12) were conducted. The multiple comparisons among the four groups were tested using Levene’s test for homogeneity of variance, and if significance was observed, Steel’s test was employed for testing, while Dunnett’s test was used when significance was not observed.

For within-group comparisons between the data at week 0 and the data at weeks 4, 8, and 12 of test food intake, multiple comparisons were conducted using Levene’s test to assess homogeneity of variance. If significance was observed, Steel’s test was employed for testing, while Dunnett’s test was used when significance was not observed.

The basic statistics of the analyzed data were calculated using Microsoft Excel for Office365 MSO (Microsoft Japan Corporation, Tokyo, Japan), and statistical processing was performed using R statistical software v4.1.0 for Windows (R Core Team, Vienna, Austria). In all significance analyses, a significance level below 5% was considered statistically significant, while a level between 5% and less than 10% was considered as a trend.

## 3. Results

### 3.1. Subjects’ Eligibility, Registration, and Flowchart

**Figure 1:**
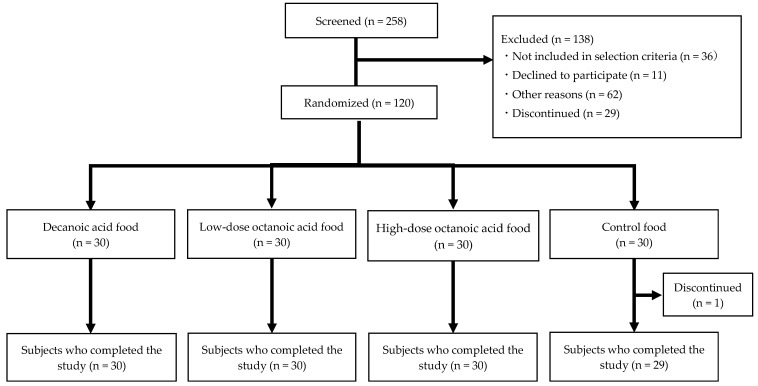
Flowchart of study subjects.

A total of 258 participants who provided consent were screened of whom 120 individuals who met the selection criteria and did not violate the exclusion criteria were enrolled as subjects. Out of the enrolled subjects, 119 completed the trial; 1 individual was excluded who discontinued participation after the start of the study. A total of 112 subjects, excluding those who did not meet the defined criteria in the statistical analysis plan, were analyzed as per protocol set (PPS) (Figure 1). The background characteristics of the analyzed subjects are presented in Table 2.

### 3.2. Adverse Events

The principal investigator evaluated presence of adverse events in the 120 registered subjects. A total of 103 adverse events were observed, but no association with the test food was determined for any of the events. Therefore, no side effects were attributed to the test food.

### 3.3. Nutrient Intake

Nutrient intake values (excluding the test food) during the study period are shown in Table 3. There were no significant differences in nutrient intake between the control and test food groups at baseline, and at week 4, week 8, and week 12 post-intervention.

### 3.4. Body Composition

The values of body composition during the study period are shown in Table 4. No significant differences were observed between the test food group and the control group in terms of body weight, protein mass, body fat mass, muscle mass, and lean body mass at baseline, and at week 4, week 8, and week 12 post-intervention.

### 3.5. Muscle Strength

At week 12 of the intervention, the knee extension strength of all MCT intake groups showed an increase compared to pre-intake values (Table 5). At the same point, the high-dose octanoic acid group and the decanoic acid group exhibited higher values compared to the control group. In terms of the change from pre-intake values, the right or bilateral knee extension strength of all MCT intake groups was higher than that of the control group. Although no intergroup differences were observed in grip strength, the left-hand maximum grip strength significantly increased from baseline in the high-dose octanoic acid group and the decanoic acid group that consumed 6 g of MCT per day.

### 3.6. Walking Ability (TUG)

Lower values indicate improved walking ability. In all groups, including the control group, there was a significant decrease compared to pre-intake values thus indicating improvement (Table 6). No significant differences were observed among the groups.

## 4. Discussion

Cross-sectional studies have found an association between low BMI and frailty [21]. In this study, the BMIs of eligible individuals were below 24 kg/m^2^ according to the inclusion criteria. As a result, the subjects generally had a BMI lower than average for their age group in the Japanese population, indicating that they were healthy middle-aged and older adults at high risk of frailty. These subjects were given MCT while engaging in moderate-intensity exercise for 12 weeks. The results showed that the knee extension strength of all MCT intervention groups significantly increased after 8 and 12 weeks compared to the pre-intervention values. Furthermore, the change in right knee extension strength from pre-intervention to week 12 was significantly higher in all MCT intervention groups compared to the control group. On the other hand, grip strength increased in the group receiving 6 g/day of MCT compared to pre-intervention, but there was no significant difference compared to the control group.

We used the Locomo Scan-II device to measure knee extension strength. The knee extension strength has been measured using an isokinetic machine as the gold standard method, but such machines are large, immovable, require a skilled operator, and are not suitable for quick measurements on a large group of subjects. Oomori et al. reported that the knee extension strength measured using the Locomo Scan-II prototype showed a significant correlation with the measurements obtained using an isokinetic dynamometer (Biodex system 3; Biodex Medical System Inc., Shirley, New York, NY, USA) in a sample of 1016 patients with knee osteoarthritis thus confirming its utility [22]. Furthermore, a study measuring knee extension strength using the Locomo Scan-II in 3617 healthy individuals aged 20–89 from 20 prefectures in Japan found no significant difference in strength among individuals aged 20–40 years, but both men and women in older age groups showed a significant decline in knee extension strength with increasing age [23].

Knee extension strength is widely used in clinical practice as a representative measure of lower limb support during walking, and it is reportedly closely related to walking independence and walking speed [24,25]. It has been suggested that older adults with problems in knee extension strength face difficulties in mobility [26]. Asakawa et al. demonstrated a close relationship between decreased knee extension strength in community-dwelling older adults and falls [27]. Moreover, measuring knee extension strength using an isokinetic machine is an accurate method for assessing quadriceps muscle strength. Many studies [26,27,28,29] have demonstrated that quadriceps muscle strength is associated with walking ability, suggesting that weakened quadriceps can lead to gait impairments and increased fall risk in older adults. Furthermore, each muscle group in the lower limbs is related to mobility limitation (ML), which affects the performance of movement activities and is associated with a decline in activities of daily living (ADL) and reduced quality of life in older adults [30]. It has been shown that reduced leg muscle strength strongly influences ML [31]. ML in older adults [32] is recognized as an early sign of impairment associated with aging stages [33]. Walking, stair climbing, and chair rising represent fundamental mobility abilities and risk factors, and a decline in these abilities is associated with poor self-rated health [34], falls [35], hospitalization [36], and overall mortality [37]. Therefore, it is crucial to identify ML early and provide appropriate support as a primary preventive measure.

In this study, we measured knee extension strength using the Locomo Scan-II device as a tool to assess the degree of age-related impairments that increase the risk of physical frailty and the effectiveness of interventions in mitigating these impairments. The average knee extension strength in the study subjects was 327 N for men and 277 N for women in their 60s, and 300 N for men and 262 N for women in their 70s. In a study by Narumi et al. that measured knee extension strength using the Locomo Scan in healthy individuals, the average values were 471 N for men and 405 N for women in their 60s, and 385 N for men and 340 N for women in their 70s [23]. Therefore, the knee extension strength of the subjects in this study was approximately 30% lower in their 60s and slightly over 20% lower in their 70s compared to the measurements by Narumi et al., suggesting that they were closer to frailty despite being healthy middle-aged and older individuals. On the other hand, the post-intake knee extension strength of the subjects in the MCT intake group was higher compared to the pre-intake or the control group. Therefore, for healthy middle-aged and older individuals at a higher risk of frailty, combining moderate-intensity exercise with MCT intake can potentially improve knee extension strength and suppress ML thereby enhancing walking independence.

In this study, grip strength was also measured. Since walking was prescribed as a moderate-intensity exercise in this trial, there was no direct stimulation to the upper limb muscles through exercise. However, ghrelin which is secreted from the lower limb muscles due to walking or from the stomach because of MCT intake may have a positive impact on the muscles related to grip strength through the stimulated secretion of IGF-1. The MCT intake group showed a significant increase in grip strength compared to the baseline. Although there was no statistically significant difference in grip strength between the MCT intake group and the control group, it is speculated that MCT intake promoted the secretion of ghrelin in the subjects’ bodies, leading to an increased secretion of IGF-1, which in turn resulted in increased grip strength compared to before MCT intake. Grip strength is related to knee extension strength [38], and it is considered a potential indicator for assessing ML. The main strengths of grip strength measurement lie in its simplicity, high reliability, and safety, as it can be measured via a simple gripping motion. A systematic review examining the association between performance tests and health outcomes [39] demonstrated that grip strength is the most widely applicable field test. Furthermore, considering its association with frailty [40], health-related quality of life [41], ADL impairments [42], and overall mortality [43], which are more advanced stages of impairment beyond ML, grip strength has a high potential to reflect the early signs of ML. 

In this study, body muscle mass was measured in healthy subjects and no significant differences were found between the groups. Similar results were reported in a previous study in which severely frail subjects with sarcopenia were fed the same amount of fats and oils with the same MCFA composition as the high octanoic acid food group in this study. Abe et al. found that after 3 months of consumption of 6 g of MCT and 1.2 g of L-leucine or 20 μg of cholecalciferol or 6 g of MCT per day by residents of a special nursing home who required helper assistance, knee extension time (a measure of muscle endurance) and the number of leg openings and closings over 10 s (a measure of muscle strength) improved significantly compared to controls. Although they improved significantly, there were no differences in the calculated AMA or leg circumference (a measure of muscle mass) between the study groups [15,19]. In previous meta-analyses, the administration of 9–54 g/day MCT was not found to be associated with increased muscle mass [44,45]. Furthermore, the administration of 18–24 g/day MCT decreased fat-free mass in obese patients in comparison with an olive oil-fed group [46], suggesting that the administration of MCT is unlikely to substantially increase muscle mass. Several studies have reported that even when muscle strength and function are enhanced through MCT intake, there are no changes in muscle mass.

The balance between muscle protein synthesis (MPS) and muscle protein breakdown (MPB) determines the net rate of muscle growth [47]. In the fasting state, there is a negative protein balance resulting in a decrease in protein mass. Feeding induces an increase in muscle protein synthesis, offsetting the losses in the fasting state. Through this cycle, muscle mass is generally maintained in adults. However, humans (typically beyond the age of 60 years) begin to experience a gradual decline in overall muscle mass, albeit at a slow rate [48]. This is caused by a gradual decrease in the rate of muscle protein synthesis with age [49]. There is limited evidence regarding the effects of MCT on muscle protein synthesis (MPS), including animal studies. Regarding the effects of MCT on muscle protein breakdown (MPB), Nishimura et al. suggested the potential inhibition of disuse muscle atrophy in soleus muscle [50]. They demonstrated that the decrease in muscle mass and muscle protein content caused by disuse muscle atrophy in rats with immobilization was suppressed due to MCT intake. It was speculated that MCT inhibits the ubiquitin-proteasome pathway, which is a major protein degradation pathway upregulated in inactive muscles. The subjects in this study had an exercise frequency of less than the average for their age group, indicating that this was a group experiencing muscle degeneration due to protein breakdown from inactivity. Although there is a possibility that MCT intake inhibited muscle protein breakdown in these subjects, there was no difference in muscle mass between the MCT intake group and the control group. This may be because the effect of MCT on human muscle protein breakdown was so small that no change in muscle mass occurred, or because changes in muscle mass take longer to occur.

The effects of MCT intake, which are speculated to be the underlying mechanisms for the observed increase in muscle strength in this study, have been reported in previous studies. For instance, Ishizawa et al. compared rats fed a high-fat diet with either LCT or MCT for 4 weeks to rats fed a standard diet. They found that MCT intake resulted in increased mitochondrial citrate synthase enzyme activity in all types of skeletal muscles examined (triceps brachii, superficial and deep portions of the gastrocnemius, and soleus muscle) compared to the control group fed a standard diet (*p* < 0.05). This suggested that MCT intake may increase the expression of citrate synthase enzymes in both fast-twitch and slow-twitch muscle fibers, promoting fatty acid oxidation [51]. Fushiki et al. conducted a study where mice underwent forced swimming every other day for 6 weeks while being fed either a LCFA diet or a MCFAs diet [52]. There was no significant difference in the weight of the quadriceps femoris or gastrocnemius muscles on the final day of the experiment. However, in the quadriceps femoris of the MCFAs diet group, the activity of 3-oxo acid CoA-transferase (OXCT) (an enzyme involved in the uptake of ketone bodies from the bloodstream), citrate synthase enzymes involved in the TCA cycle in the gastrocnemius, and malate dehydrogenase were significantly higher than in the LCFA diet group. This suggested an increased rate of uptake and utilization of ketone bodies (a lipid-based energy substrate) by muscle cells, and enhanced intracellular energy production. The swimming capacity of these mice, until exhaustion, was significantly higher in the MCFAs diet group compared to the LCFA diet group. It is speculated that the changes induced by MCFAs in these enzymes contributed to the increased swimming capacity. However, there was no change in the weight of the muscles despite the alterations in enzyme activity. In the subjects of our study, the impact of MCT intake on muscle protein metabolism was not significant enough to cause a significant difference in muscle mass compared to the control group. However, the changes in energy metabolism enzymes in the muscles likely enhanced fatty acid oxidation capacity, resulting in abundant energy supply during muscle activation and an increase in muscle strength. Therefore, MCT may act on muscle energy metabolism rather than muscle protein metabolism.

There have been several reports on the effects of combining exercise with food components other than MCT on muscle mass, muscle strength, and muscle function in healthy older adults. Nagai et al. reported the results of a randomized controlled trial (RCT) with resistance training, in which 36 healthy men and women aged 71 to 76 years assigned to either a control group (*n* = 19) or an intervention group (*n* = 17) received 60 mg/day of maslinic acid or a placebo for 12 weeks [53]. The 12-week maslinic acid intervention significantly improved skeletal muscle mass and segmental muscle mass (right arm, left arm, and trunk). Additionally, only the intervention group showed a significant increase in grip strength. In addition, Vukovich et al. reported the results of an RCT with resistance training, where 31 healthy men and women aged 70 ± 1 years were assigned to either a control group (*n* = 17) or an intervention group (*n* = 14) received 3 g/day of β-hydroxy-β-methylbutyrate (HMB) or a placebo for 8 weeks [54]. The intervention group showed a tendency of increased fat-free mass after 8 weeks compared to the control group (*p* = 0.08). Additionally, the intervention group exhibited a significant increase in leg strength compared to the control group after 8 weeks. Furthermore, Stout et al. [55] reported the results of an RCT with resistance training, in which 36 healthy men and women aged 65 years and older were assigned to either a control group (*n* = 20) or an intervention group (*n* = 16) received 3 g/day of calcium β-hydroxy-β-methylbutyrate (CaHMB) or a placebo for 24 weeks. Although there were no significant differences between the control and intervention groups, both groups showed a significant increase (*p* < 0.05) in muscle mass and muscle strength (leg strength) after 24 weeks compared to the baseline.

In these interventions, unlike MCT, there were differences in muscle mass compared to the control groups. As a result, it is suggested that muscle mass was maintained. One possible reason for the higher muscle mass is the potential effect of these food components on stimulating muscle protein synthesis. For instance, when maslinic acid was administered in the feed of rainbow trout for 225 days, a significant increase in white muscle weight and protein synthesis rate was observed in the trout compared to the control group [56]. Moreover, the addition of maslinic acid to C2C12 myoblast cells resulted in enhanced phosphorylation of the 70 kDa ribosomal protein S6 kinase (p70S6K), downstream of mammalian target of rapamycin (m-TOR), leading to increased protein synthesis [57]. In mice induced with disuse muscle atrophy, oral administration of maslinic acid increased protein synthesis in the gastrocnemius muscle, and continuous intake of maslinic acid for 14 days significantly increased muscle weight in the gastrocnemius and soleus muscles, as well as grip strength [58]. Based on the above, it is thought that maslinic acid intake may be effective in maintaining muscle mass, which decreases with age, as it improves muscle protein synthesis function through the activation of m-TOR. On the other hand, the involvement of HMB and CaHMB increases phosphorylation of mammalian target of rapamycin protein (hereafter referred to as m-TOR), its downstream 70 kDa ribosomal protein S6 kinase (p70S6K), and eukaryotic translation initiation factor-4 binding protein-1 (4E-BP1) (which constituent 3-hydroxy-3-methylbutyrate). It may promote muscle protein synthesis by directly activating m-TOR, which plays a central role in cell growth [59,60,61,62]. In addition, GH and IGF-1, which are upstream of m-TOR, are increased, suggesting that m-TOR and muscle protein synthesis are activated through changes in GH/IGF-1 activity [63,64,65]. In other words, food components that promote muscle protein synthesis, such as maslinic acid and HMB, are thought to contribute to the maintenance of muscle mass in the elderly by suppressing the decline in MPS and the imbalance between MPS and MPB. Additionally, while studies on maslinic acid and HMB have employed resistance exercise as a concurrent exercise, this study involved moderate-intensity exercise. This difference in exercise type may be one factor contributing to the discrepancy in results between this study and interventions with maslinic acid and HMB.

In this study, the results showed that while there was no significant improvement in muscle mass, there was a significant improvement in muscle strength after 12 weeks of concurrent intake of MCT along with moderate-intensity exercise. Additionally, it is generally believed that muscle strength does not improve with training below moderate intensity. However, in this study, the control group that consumed LCT showed an increase in muscle strength after 12 weeks, and the MCT group demonstrated a significantly greater increase in muscle strength compared to the LCT group.

Resistance training has been considered the most effective exercise therapy for maintaining and improving muscle mass in patients with sarcopenia. Typically, strength training with a load setting of 60–70% of one-repetition maximum (1RM) has been recommended for the purpose of muscle hypertrophy and strength enhancement [66]. For instance, Balakrishnan et al. observed an increase in mitochondrial DNA copy number in the external oblique muscle of elderly CKD patients with mitochondrial dysfunction following resistance training load. There was a positive correlation between changes in mitochondrial DNA copy number, type I and type II muscle fiber cross-sectional area, and muscle strength [67]. These findings suggest that interventions promoting mitochondrial biogenesis, such as resistance training, may be associated with increased muscle strength. However, exercise training is adopted only by a very small percentage of elderly individuals, possibly because of their frailty (15]. Furthermore, some reports suggest that resistance training is contraindicated in malnutrition [68]. Therefore, caution should be exercised when applying resistance training to the elderly for the purpose of maintaining muscle mass [69].

Recently, it has become evident that muscle hypertrophy can be induced by repeatedly performing long-duration low-intensity exercise to the point of fatigue [70]. A study conducted among community-dwelling individuals aged 60 years and above, involving 12 weeks of low-intensity exercise (20% of 1RM) with high frequency (1 set of 80–100 repetitions), resulted in a significant increase in muscle mass [71]. Furthermore, moderate-intensity exercise load has the potential to improve mitochondrial dysfunction, a factor contributing to sarcopenia due to its activation of peroxisome proliferators-activated receptor-γ co-activator-1α (PGC1-α) and increased mitochondrial biogenesis [72,73]. Therefore, even with exercise interventions of lower intensity than resistance training, it has been suggested that sufficient energy supply to skeletal muscles can enhance muscle function. In fact, reports have shown that long-term implementation of endurance training such as walking leads to improvements in muscle strength and hypertrophic effects [70]. Additionally, while exercise intervention alone may not have significant effects on muscle mass and strength, some reports indicate that combining it with dietary therapy can enhance its effectiveness. For instance, Yamada et al., conducted an RCT comparing a resistance training plus nutrition group with a resistance training alone group among frail elderly individuals, and reported that the resistance training plus nutrition group showed significant improvements in skeletal muscle index, maximum walking speed, and a decrease in the prevalence of sarcopenia, whereas the resistance training alone group remained unchanged [74].

We investigated the possibility of maintaining muscle mass and strength with a combination of dietary and exercise interventions, even with lower intensity exercise. As a result, lower limb strength increased, but it did not lead to an increase in muscle mass. This could be attributed to factors such as the intensity or frequency of the exercise employed in this study, or the duration of the intervention, which may have been insufficient compared to previous reports that demonstrated changes in muscle mass with exercise loads below moderate intensity. On the other hand, even in the control group that consumed LCT, knee extension strength increased compared to the pre-intervention measurements. In middle-aged and older individuals who are at high risk of future frailty due to lack of exercise or a sedentary lifestyle, it is believed that, if they are healthy, resolving the issue of physical inactivity through exercise can lead to the maintenance of muscle strength and reduction in frailty risk. The results suggest that MCT intake has the potential to further enhance the preventive effects against frailty.

In this study, we also examined the effects of different quantities and compositions of MCFAs in MCT. Regarding the observed changes and trends in knee extension strength and grip strength in this study, we investigated the differences in the composition of MCFAs in MCT consumed. However, no significant differences were found between the group consuming MCT with a high dose of octanoic acid (6 g/day) and the group consuming MCT with decanoic acid. The daily intake of MCFAs in each group was as follows: for the decanoic acid group, octanoic acid ranged from 1.40 to 1.95 g and decanoic acid ranged from 3.47 to 4.00 g; for the high-dose octanoic acid group, octanoic acid ranged from 3.72 to 4.14 g and decanoic acid ranged from 1.06 to 1.46 g; and for the low-dose octanoic acid group, octanoic acid ranged from 1.24 to 1.38 g and decanoic acid ranged from 0.352 to 0.487 g. The high-dose octanoic acid group and low-dose octanoic acid group predominantly contained octanoic acid, while the decanoic acid group contained decanoic acid as the major fatty acid. Although all are MCFAs, they reportedly have different physiological effects. In studies using in vitro or type 2 diabetes model animals, a mixture of octanoic acid and decanoic acid has shown potential to protect islet β-cell function from impairment [18]. Octanoic acid directly induced ketone body synthesis in mitochondria, while decanoic acid promoted ketone body production in β-cells through activation of the fatty acid receptor GPR40. It is believed that the resulting β-hydroxybutyric acid improved beta cell function and alleviated insulin resistance.

With regard to energy supply in skeletal muscles, it has been suggested that octanoic acid increases the supply of energy sources to muscles through ketone body production in the liver, while decanoic acid has the potential to increase mitochondrial biogenesis and energy production in skeletal muscles through the peroxisome proliferator-activated receptor (PPAR), which is a fatty acid receptor. Fukazawa et al. reported an increased protein expression of OXCT (a ketone body-utilizing enzyme) in the epitrochlearis muscle of rats fed a ketogenic high-fat diet containing octanoic acid-dominant MCT, similar to the fat intake of the high-dose octanoic acid food intake group in this study [75]. Similar changes were observed in the ketogenic LCT high-fat diet group, but the OXCT expression level was lower than that of the MCT intake group. Thus, it is inferred that octanoic acid leads to increased ketone body production in the liver after ingestion, and these ketone bodies are transported to the muscles through the bloodstream where they are utilized as energy substrates. Octanoic acid has also been shown to activate ghrelin. When mice were fed MCT composed of octanoic acid, the amount of octanoyl-ghrelin (the active form of ghrelin) was significantly higher compared to the control group. However, there was no significant difference observed between the control group and mice fed MCT composed of decanoic acid [16]. Ghrelin strongly promotes the secretion of GH, which in turn stimulates skeletal muscle protein synthesis through IGF-1. Administration of ghrelin to mice with disuse muscle atrophy reportedly suppresses muscle loss in both fast-twitch and slow-twitch muscles [76]. Additionally, in malnourished individuals with chronic respiratory diseases, it was shown that plasma levels of active ghrelin and IGF-1 significantly increased after 2 weeks of daily administration of 3 g of MCT [17]. Furthermore, a correlation has been observed between the intake of MCT (<1 g/day, 1–6 g/day, and >6 g/day) and the levels of active ghrelin in patients with anorexia nervosa [77]. On the other hand, among the PPAR family (which are nuclear receptors regulating mitochondrial biogenesis and beta-oxidation, and are controlled by various fatty acids), the activation ability of PPARα (localized in the liver) by decanoic acid is significantly higher than that of octanoic acid [78]. Additionally, it has been demonstrated that PPARγ, which is abundant in skeletal muscle, is directly activated by decanoic acid as a ligand [79]. In this case, even at high concentrations, octanoic acid does not show significant binding to PPARγ. Therefore, it is speculated that decanoic acid primarily plays a role in the intracellular metabolism regulation of MCFAs through PPARγ, rather than octanoic acid. Based on the above findings, it is possible that octanoic acid and decanoic acid have the potential to improve skeletal muscle energy utilization through different mechanisms. In this study, it appears that the effects of each fatty acid on changes in muscle strength were comparable. As a result, it is presumed that the changes and trends in knee extension muscle strength and grip strength were similar between the high-dose octanoic acid food intake group and the decanoic acid food intake group.

In this study, although the changes in knee extension muscle strength were similar among the two groups that consumed 6 g of MCT and the group that consumed 2 g of low-dose octanoic acid food, the changes in grip strength were different. The maximum left-hand grip strength in the 6 g intake group showed no significant difference compared to the control group, but a significant increase was observed compared to the baseline. On the other hand, the maximum left-hand grip strength in the low-dose octanoic acid food intake group did not show significant changes compared to the baseline throughout the intake period. Tsujino et al. reported that ingestion of 2 g of MCT, predominantly composed of octanoic acid, for 2 weeks in 29 healthy individuals with an average age of 50.3 years (17 males and 12 females) resulted in an increased rate of fatty acid oxidation during daily activities [80]. They speculated that mitochondrial biogenesis increased and metabolism-related enzymes were activated in the bodies of subjects who continued to consume MCT, leading to an increase in skeletal muscle fatty acid oxidation. If mitochondrial biogenesis is increased, as in the study by Balakrishnan et al. [67], an increase in muscle strength can also be expected. It is speculated that similar changes occurred in the bodies of the subjects in this study, and even with a small amount of 2 g of MCT, compared to the two experiments by Abe et al. [15,19] (which targeted older adults who were administered 6 g of MCT), changes in knee extension muscle strength may have occurred due to the additive effect of direct stimulation to the lower limb muscles through walking. On the other hand, the effect of a small amount of MCT on the upper arm (where there is no direct exercise stimulus due to the intervention) may be insufficient to induce changes in grip strength, which is why no significant changes were observed.

As mentioned above, MCT did not induce changes in muscle mass in this study, and previous evidence has also not observed favorable changes in muscle mass or lean body mass with MCT intake, suggesting a limited effect on skeletal muscle protein metabolism. However, there have been reports of potential effects on liver protein metabolism. Administration of MCT to protein–energy malnutrition (PEM) model animals fed a low-protein diet resulted in enhanced protein synthesis pathways in the liver and increased liver albumin levels. As a result, serum albumin levels were also higher compared to the LCT intake group [81]. Similar effects have been observed in humans. In undernourished long-term hospitalized patients (with an average age of 80 years) given 6 g of MCT per day for 12 weeks, serum albumin levels increased compared to baselines and the LCT intake group [82]. Since malnutrition is a risk factor for sarcopenia, the improvement in serum albumin (a marker of nutritional status) is believed to contribute to reducing the risk of sarcopenia, similar to the changes observed in muscles.

Recently, Hirabayashi et al. induced sarcopenia in model animals via low-protein diet restriction and administration of MCT-containing food during the recovery period. As a result, increased phosphorylation of rpS6 in the liver and tibialis anterior muscle was observed, along with a higher muscle fiber protein content and cross-sectional area compared to the group that received an LCT-containing diet during the recovery period [83]. Kimura et al. reported that in the gastrocnemius muscle of a primary sarcopenia model animal fed with MCT, the mRNA expression of Myh3, which is involved in muscle regeneration from satellite cells, was significantly higher compared to the LCT-fed group, while the mRNA expression of Murf1 (a factor that promotes muscle breakdown in muscle cells) was significantly lower [84]. These findings suggest that appropriately implementing a dietary therapy using MCT may potentially increase muscle mass, which was not observed in the present study.

In this study, the TUG was conducted to measure walking ability. TUG is highly correlated with lower limb strength, balance, walking ability, and ADL, and it reportedly has good reliability and validity [20,85,86]. Moreover, it is considered beneficial as a physical function assessment tool for community health activities in older adults, and it can also be used as a screening test to predict fall risk [87], with a cutoff value of 13.5 s suggested as an indicator of fall risk. The decline in walking ability in older adults restricts their mobility, leading to decreased ADL and QOL [88,89], and it is also associated with institutionalization and mortality [29,90]. Therefore, it is important to appropriately assess walking function for maintaining the health of older adults. In this study, while the TUG time suggested a significant improvement in walking ability compared to baseline in both the MCT group and the control group, no significant difference was detected between the groups in terms of TUG time. This may be attributed to the fact that the control group was also subjected to moderate-intensity walking, leading to an improvement in walking function through enhancements in lower limb strength and balance. Similar findings were reported by Stout et al., where the effects of dietary components were nullified by exercise load [55]. They reported the results of two RCTs involving healthy men and women aged 65 years and above, with one RCT not incorporating resistance training and the other RCT incorporating resistance training. In the RCT without resistance training, the intervention group showed significant increases in muscle mass and muscle strength compared to baseline after 24 weeks, while the control group showed no significant changes. Furthermore, when compared to the control group, the intervention group exhibited a significant increase in muscle strength after 24 weeks (*p* = 0.04). On the other hand, in the RCT incorporating resistance training, both the control and intervention groups showed significant increases in muscle mass and muscle strength compared to baseline after 24 weeks (*p* < 0.05), resulting in no significant difference between the control and intervention groups.

The results of the TUG also reflect balance function during walking. In this study, although no significant difference was observed between the groups in terms of static balance function measurement, the effects of MCT on balance function during walking have been reported. Mutoh et al. reported that healthy older adults with an average age of 69.8 years who consumed 18 g of MCT per day for 3 months showed improved balance during walking compared to the control group [91]. Previous studies have demonstrated that age-related impairment in proprioceptive sensation affects balance control during walking [92]. When measuring brain glucose utilization in 40 healthy individuals aged 18 to 78 years, divided into eight age groups, a gradual decline in glucose utilization was reported with increasing age [93]. Moreover, individuals with early stage dementia reportedly show significantly lower brain glucose utilization compared to healthy individuals [94], suggesting that the age-related decline in brain glucose utilization may be associated with impaired brain function, including balance control. In studies that administered MCFAs to type 1 diabetes patients to induce cognitive impairment by forcibly lowering blood glucose levels and intravenously administered ketone bodies to healthy individuals to induce cognitive impairment using the same method, a significant reduction in cognitive impairment was observed compared to control groups that did not receive MCT or ketone bodies [95,96]. In the study by Mutoh et al., it is possible that the MCFAs derived from the consumed MCT directly or through the generated ketone bodies enhanced brain function and contributed to the improvement in balance during walking. In our current study, it is also possible that the consumed MCT enhanced brain function, thereby improving balance. Although there was no significant difference in the change in TUG values between the groups in our study, the MCT group generally showed a greater decrease in the required time and a larger improvement in walking function compared to the control group. This may be attributed to the improvement in balance function through MCT consumption, which is closely related to walking function, in addition to the improvement in knee extension strength.

No adverse events attributable to the ingestion of the test foods were observed in this study. However, adverse events related to both single-dose intake [97] and continuous intake [98] of MCT have been reported. However, in the present study, no subjects reported discomfort, and no adverse events related to MCT intake were observed. Based on these findings, it was concluded that no adverse effects were manifested with the continuous intake of MCT. Moreover, the intake rate of MCT-containing test food among the subjects during the study period was over 98% for all subjects, indicating high acceptability in terms of taste, flavor, and other aspects, making it suitable for continuous intake over a period of 3 months.

In this study, walking was imposed as the exercise load, which is relatively easy to engage in. However, it is worth noting that there are many older adults who have impairments in their lower limbs. The effects of MCT intake on muscle strength in such individuals, when combined with different forms of exercise load, remain unknown. To clarify the effects of MCT on age-related muscle degeneration and reduced mobility in middle-aged and older individuals, a large-scale intervention study may be necessary. In addition, the subjects’ residential areas were limited. This study was conducted with healthy middle-aged and older adults living at home in urban areas. In rural areas, compared to urban areas, there tends to be a smaller number of destinations (such as restaurants and retail stores), and fewer opportunities for older adults to engage in group activities, hobbies, sports, and so on. Furthermore, public transportation in rural areas of Japan has fewer services and is less convenient. As a result, individuals in rural areas may have fewer opportunities for outdoor activities. Social isolation and confinement at home are considered one of the most important risk factors associated with functional decline and the development of mobility and instrumental activities of daily living (IADL) impairments [99]. Tsubokawa et al. investigated the relationship between homebound status and the surrounding environment in older adults in Niigata Prefecture, which has both typical urban areas and mountainous rural areas in Japan. Their study demonstrated that the presence of parks and sidewalks suitable for walking and exercise, more commonly found in urban areas, could prevent the homebound status among older adults [100]. Thus, the subjects in this study had lower physical decline compared to individuals of the same age living in rural areas. If a similar study were conducted in suburban or rural areas (recruiting middle-aged and older adults of similar age, activity level, and physique as the subjects in this study), the frailty risk of that group may be different. Therefore, the effectiveness of MCT on healthy middle-aged and older adults across Japan, including suburban and rural areas, remains unknown.

## 5. Conclusions

This study revealed that the combination of moderate-intensity exercise, such as walking, and continuous intake of MCT composed exclusively of MCFAs, is more effective in maintaining and suppressing the decline of skeletal muscle strength compared to the regular intake of LCT alone. In other words, it has been demonstrated that the combination of a relatively easy-to-implement exercise therapy without resistance training and MCT intake shows additional preventive effects. It is speculated that the action of ingested MCFAs enhances fatty acid oxidation capacity in the muscles, resulting in increased muscle strength compared to LCT intake, as it provides ample energy supply during muscle activation. The increase in muscle strength through the intake of foods containing MCFAs and the implementation of an active lifestyle contributes to the maintenance of health in middle-aged and older adults at high risk of frailty and, consequently, achieves societal contributions through the reduction in national medical and nursing care expenses by extending healthy life expectancy.

## Figures and Tables

**Table 1:** Content of LCT, MCT and MCFAs of MCTs in 6 g of oil in the test diet.

		Control Food	Decanoic Acid Food	Low-Dose Octanoic Acid Food	High-Dose Octanoic Acid Food
LCT	g/day ^1^	6	-	4	-
MCT	g/day ^1^	-	6	2	6
Octanoic acid (8:0)	g/day ^2^	(-)	(1.40, 1.95)	(1.24, 1.38)	(3.72, 4.14)
Decanoic acid (10:0)	g/day ^2^	(-)	(3.47, 4.00)	(0.352, 0.487)	(1.06, 1.46)

^1^ Values show the amount of content. ^2^ Lower and upper limits are shown.

**Table 2:** Characteristics information of the PPS ^1^ subjects ^2^.

	PPS (*n* = 112)			
	Control Food	Decanoic Acid Food	Low-Dose Octanoic Acid Food	High-Dose Octanoic Acid Food
Number of subjects, M/F	29 (14/15)	27 (12/15)	28 (13/15)	28 (15/13)
Age, years	67.6 ± 0.8	67.9 ± 0.8	68.2 ± 0.8	67.7 ± 0.8
Height, cm	160.2 ± 1.3	161.4 ± 1.5	159.6 ± 1.7	163.4 ± 1.5
Blood albumin level, g/dL	4.5 ± 0.0	4.6 ± 0.1	4.6 ± 0.1	4.5 ± 0.1

^1^ Per protocol set: dataset of subjects excluding those who were excluded as per the defined criteria in the statistical analysis plan. ^2^ Values are shown as mean ± standard deviation.

**Table 3:** Nutrient intakes during the intervention period (excluding the nutrient from test food) ^1,2^.

		PPS ^3^ (*n* = 112)			
	Week	Control Food	Decanoic Acid Food	Low-Dose Octanoic Acid Food	High-Dose Octanoic Acid Food
Energy, kcal/day	0	1600.4 ± 104.7	1585.3 ± 75.7	1572.0 ± 80.7	1710.6 ± 92.5
	4	1598.6 ± 106.3	1594.4 ± 76.3	1578.3 ± 74.3	1733.3 ± 95.6
	8	1583.3 ± 99.4	1570.1 ± 85.4	1553.3 ± 83.4	1776.7 ± 82.1
	12	1563.7 ± 101.5	1552.2 ± 76.5	1579.8 ± 70.1	1717.7 ± 91.2
Fat, g/day	0	49.9 ± 3.6	51.9 ± 3.6	53.5 ± 2.6	53.4 ± 3.7
	4	49.9 ± 3.7	52.8 ± 3.4	53.0 ± 2.8	54.1 ± 3.8
	8	47.9 ± 3.2	54.1 ± 3.6	52.5 ± 3.1	55.7 ± 3.8
	12	48.8 ± 3.5	51.0 ± 3.4	53.8 ± 2.7	54.4 ± 3.8
Saturated fatty acid, g/day ^4^	0	12.8 ± 1.1	13.5 ± 0.9	13.7 ± 0.7	14.0 ± 1.1
	4	12.8 ± 1.1	13.7 ± 0.9	13.4 ± 0.8	14.2 ± 1.1
	8	12.5 ± 1.0	14.0 ± 1.0	13.4 ± 0.8	14.3 ± 1.1
	12	12.6 ± 1.1	13.8 ± 0.8	13.8 ± 0.8	14.2 ± 1.1
Monounsaturated fatty acid, g/day	0	17.9 ± 1.3	18.5 ± 1.4	19.0 ± 1.0	18.9 ± 1.4
	4	17.9 ± 1.3	18.9 ± 1.3	18.8 ± 1.1	19.2 ± 1.4
	8	17.0 ± 1.2	19.4 ± 1.4	18.6 ± 1.1	19.8 ± 1.5
	12	17.4 ± 1.3	18.1 ± 1.3	19.1 ± 1.0	19.3 ± 1.4
n-6 Polyunsaturated fatty acid, g/day	0	9.9 ± 0.6	10.4 ± 0.7	10.7 ± 0.6	10.5 ± 0.7
	4	9.9 ± 0.6	10.6 ± 0.7	10.8 ± 0.6	10.7 ± 0.7
	8	9.5 ± 0.6	10.9 ± 0.7	10.4 ± 0.6	11.1 ± 0.7
	12	9.7 ± 0.6	10.1 ± 0.7	10.8 ± 0.6	10.7 ± 0.7
n-3 Polyunsaturated fatty acid, g/day	0	2.4 ± 0.2	2.5 ± 0.2	2.6 ± 0.2	2.6 ± 0.2
	4	2.4 ± 0.2	2.5 ± 0.2	2.7 ± 0.2	2.6 ± 0.2
	8	2.2 ± 0.2	2.5 ± 0.2	2.7 ± 0.3	2.8 ± 0.2
	12	2.3 ± 0.2	2.5 ± 0.2	2.6 ± 0.2	2.7 ± 0.2
Medium chain fatty acid, mg/day ^5^	0	259.0 ± 43.8	316.9 ± 43.8	289.1 ± 38.0	309.0 ± 51.4
	4	254.5 ± 43.9	316.8 ± 43.0	283.3 ± 29.3	318.2 ± 53.4
	8	265.5 ± 42.0	318.8 ± 44.4	296.5 ± 44.0	307.3 ± 44.5
	12	265.8 ± 43.4	334.4 ± 44.4	299.6 ± 39.1	313.4 ± 49.9
Protein, g/day	0	61.5 ± 3.9	63.9 ± 3.9	66.9 ± 3.6	70.5 ± 3.9
	4	61.9 ± 4.0	64.1 ± 3.8	66.5 ± 3.9	71.6 ± 4.4
	8	62.4 ± 3.7	63.3 ± 3.9	66.3 ± 4.2	71.9 ± 4.1
	12	59.6 ± 3.7	63.4 ± 3.9	66.7 ± 3.7	72.2 ± 4.4
Carbohydrate, g/day	0	212.8 ± 15.0	197.2 ± 8.2	193.6 ± 12.2	217.0 ± 13.3
	4	212.3 ± 15.2	196.2 ± 9.2	198.6 ± 10.2	220.0 ± 13.3
	8	212.6 ± 14.7	190.4 ± 9.9	190.5 ± 13.7	228.0 ± 11.4
	12	208.6 ± 14.6	194.9 ± 9.2	196.1 ± 9.9	215.6 ± 12.8
Cholesterol, mg/day	0	372.4 ± 35.3	353.5 ± 31.3	403.9 ± 31.9	432.8 ± 44.6
	4	369.2 ± 35.7	361.6 ± 29.9	400.0 ± 32.5	436.2 ± 46.4
	8	367.9 ± 33.6	368.2 ± 34.1	404.4 ± 38.4	431.9 ± 42.2
	12	354.0 ± 32.1	349.4 ± 31.1	398.3 ± 31.8	444.2 ± 46.1
Alcohol, g/day	0	5.1 ± 1.5	8.4 ± 3.1	5.1 ± 2.2	8.9 ± 2.0
	4	4.9 ± 1.4	8.9 ± 3.4	4.0 ± 1.7	8.8 ± 2.0
	8	5.1 ± 1.5	7.8 ± 2.8	5.4 ± 2.0	8.1 ± 2.1
	12	4.8 ± 1.4	6.5 ± 2.7	4.4 ± 1.9	8.3 ± 1.9

^1^ Values are shown as mean ± standard deviation. ^2^ Values are calculated using BDHQ: brief-type self-administered diet history questionnaire, a simplified self-administered dietary history questionnaire. ^3^ Per protocol set: dataset of subjects excluding those who were excluded as per the defined criteria in the statistical analysis plan. ^4^ Values were shown minus the value of octanoic acid and decanoic acid. ^5^ Values are the sum of octanoic acid and decanoic acid (medium-chain fatty acids).

**Table 4:** The body composition values of each group during the intervention period and their changes from the baseline ^1^.

		Control Food		Decanoic Acid Food		Low-Dose Octanoic Acid Food		High-Dose Octanoic Acid Food	
	Week	Measurement Values	Change Values	Measurement Values	Change Values	Measurement Values	Change Values	Measurement Values	Change Values
Body weight, kg	0	56.4 ± 1.3		56.1 ± 1.6		55.3 ± 1.6		57.3 ± 1.3	
	4	56.1 ± 1.4	−0.2 ± 0.1	56.1 ± 1.5	0.0 ± 0.1	55.2 ± 1.6	−0.1 ± 0.1	57.4 ± 1.3	0.1 ± 0.1
	8	56.0 ± 1.4	−0.4 ± 0.2	55.8 ± 1.5	−0.3 ± 0.1	55.2 ± 1.5	−0.1 ± 0.1	57.2 ± 1.3	−0.1 ± 0.2
	12	55.7 ± 1.3	−0.7 ± 0.2	55.8 ± 1.5	−0.3 ± 0.2	54.9 ± 1.6	−0.5 ± 0.1	56.9 ± 1.3	−0.4 ± 0.2
Protein mass, kg	0	8.3 ± 0.2		8.2 ± 0.3		8.4 ± 0.3		8.8 ± 0.3	
	4	8.3 ± 0.2	0.0 ± 0.0	8.2 ± 0.3	0.0 ± 0.0	8.4 ± 0.3	0.0 ± 0.0	8.8 ± 0.3	0.0 ± 0.0
	8	8.2 ± 0.2	−0.1 ± 0.0	8.2 ± 0.3	−0.1 ± 0.0	8.3 ± 0.3	−0.1 ± 0.0	8.8 ± 0.2	−0.1 ± 0.0
	12	8.2 ± 0.2	−0.1 ± 0.0	8.2 ± 0.3	0.0 ± 0.0	8.3 ± 0.3	−0.1 ± 0.0	8.8 ± 0.3	0.000
Body fat mass, kg	0	14.0 ± 0.7		14.1 ± 0.7		12.4 ± 0.6		12.3 ± 0.7	
	4	13.6 ± 0.7	−0.4 ± 0.1	14.0 ± 0.6	−0.1 ± 0.2	12.2 ± 0.6	−0.2 ± 0.2	12.2 ± 0.7	0.0 ± 0.2
	8	14.0 ± 0.7	0.0 ± 0.1	14.0 ± 0.6	−0.1 ± 0.2	12.6 ± 0.6	0.1 ± 0.1	12.3 ± 0.7	0.0 ± 0.2
	12	13.5 ± 0.6	−0.5 ± 0.2	13.7 ± 0.6	−0.3 ± 0.2	12.3 ± 0.6	−0.2 ± 0.2	11.9 ± 0.7	−0.3 ± 0.3
Muscle mass, kg	0	39.9 ± 1.2		39.6 ± 1.5		40.3 ± 1.5		42.4 ± 1.2	
	4	40.0 ± 1.2	0.1 ± 0.1	39.7 ± 1.4	0.1 ± 0.2	40.5 ± 1.5	0.1 ± 0.2	42.5 ± 1.2	0.1 ± 0.1
	8	39.5 ± 1.2	−0.4 ± 0.2	39.4 ± 1.4	−0.2 ± 0.1	40.1 ± 1.5	−0.2 ± 0.2	42.3 ± 1.2	−0.2 ± 0.2
	12	39.8 ± 1.2	−0.1 ± 0.2	39.6 ± 1.5	0.0 ± 0.2	40.1 ± 1.5	−0.2 ± 0.2	42.4 ± 1.2	0.0 ± 0.2
Lean body mass, kg	0	42.4 ± 1.2		42.1 ± 1.5		42.9 ± 1.6		45.1 ± 1.3	
	4	42.5 ± 1.2	0.1 ± 0.2	42.2 ± 1.5	0.1 ± 0.2	43.0 ± 1.6	0.1 ± 0.2	45.2 ± 1.3	0.1 ± 0.2
	8	41.9 ± 1.2	−0.4 ± 0.2	41.9 ± 1.5	−0.2 ± 0.1	42.6 ± 1.5	−0.3 ± 0.2	44.9 ± 1.2	−0.2 ± 0.2
	12	42.2 ± 1.2	−0.2 ± 0.2	42.1 ± 1.5	0.0 ± 0.2	42.6 ± 1.6	−0.3 ± 0.2	45.0 ± 1.3	0.0 ± 0.2
Skeletal muscle mass, kg	0	22.9 ± 0.7		22.8 ± 0.9		23.2 ± 0.9		24.6 ± 0.8	
	4	23.0 ± 0.7	0.1 ± 0.1	22.8 ± 0.9	0.0 ± 0.1	23.3 ± 0.9	0.1 ± 0.1	24.6 ± 0.8	0.0 ± 0.1
	8	22.7 ± 0.7	−0.3 ± 0.1	22.7 ± 0.9	−0.2 ± 0.1	23.0 ± 0.9	−0.2 ± 0.1	24.4 ± 0.7	−0.2 ± 0.1
	12	22.8 ± 0.7	−0.1 ± 0.1	22.8 ± 0.9	−0.1 ± 0.1	23.1 ± 0.9	−0.2 ± 0.1	24.5 ± 0.8	−0.1 ± 0.1
Segmental muscle mass									
Right arm, kg	0	2.0 ± 0.1		2.0 ± 0.1		2.0 ± 0.1		2.2 ± 0.1	
	4	2.0 ± 0.1	0.0 ± 0.0	2.0 ± 0.1	−0.1 ± 0.0	2.0 ± 0.1	0.0 ± 0.0	2.2 ± 0.1	0.0 ± 0.0
	8	2.0 ± 0.1	0.0 ± 0.0	2.0 ± 0.1	−0.1 ± 0.0	2.0 ± 0.1	0.0 ± 0.0	2.2 ± 0.1	0.0 ± 0.0
	12	2.0 ± 0.1	0.0 ± 0.0	2.0 ± 0.1	0.0 ± 0.0	2.0 ± 0.1	0.0 ± 0.0	2.2 ± 0.1	0.0 ± 0.0
Left arm, kg	0	2.0 ± 0.1		2.0 ± 0.1		2.0 ± 0.1		2.2 ± 0.1	
	4	2.0 ± 0.1	0.0 ± 0.0	2.0 ± 0.1	−0.1 ± 0.0	2.0 ± 0.1	0.0 ± 0.0	2.2 ± 0.1	0.0 ± 0.0
	8	2.0 ± 0.1	0.0 ± 0.0	2.0 ± 0.1	−0.1 ± 0.0	2.0 ± 0.1	0.0 ± 0.0	2.2 ± 0.1	0.0 ± 0.0
	12	2.0 ± 0.1	0.0 ± 0.0	2.0 ± 0.1	−0.1 ± 0.0	2.0 ± 0.1	0.0 ± 0.0	2.2 ± 0.1	0.0 ± 0.0
Trunk, kg	0	18.1 ± 0.6		18.2 ± 0.7		18.1 ± 0.7		19.4 ± 0.6	
	4	18.1 ± 0.6	0.0 ± 0.1	17.9 ± 0.7	−0.3 ± 0.1	18.0 ± 0.7	−0.1 ± 0.1	19.3 ± 0.6	−0.2 ± 0.1
	8	17.9 ± 0.6	−0.2 ± 0.1	17.9 ± 0.7	−0.3 ± 0.1	17.9 ± 0.6	−0.2 ± 0.1	19.2 ± 0.6	−0.3 ± 0.1
	12	17.9 ± 0.6	−0.2 ± 0.1	17.9 ± 0.7	−0.3 ± 0.1	17.8 ± 0.7	−0.2 ± 0.1	19.2 ± 0.6	−0.2 ± 0.1
Right leg, kg	0	6.6 ± 0.2		6.5 ± 0.3		6.7 ± 0.3		7.0 ± 0.2	
	4	6.6 ± 0.2	0.0 ± 0.0	6.7 ± 0.3	0.2 ± 0.0	6.8 ± 0.3	0.1 ± 0.0	7.2 ± 0.2	0.1 ± 0.0
	8	6.6 ± 0.2	0.1 ± 0.0	6.6 ± 0.3	0.1 ± 0.0	6.7 ± 0.3	0.0 ± 0.0	7.2 ± 0.2	0.1 ± 0.0
	12	6.7 ± 0.2	0.1 ± 0.0	6.7 ± 0.3	0.2 ± 0.0	6.7 ± 0.3	0.1 ± 0.0	7.1 ± 0.3	0.1 ± 0.0
Left leg, kg	0	6.6 ± 0.2		6.5 ± 0.3		6.6 ± 0.3		7.0 ± 0.2	
	4	6.6 ± 0.2	0.0 ± 0.0	6.6 ± 0.3	0.2 ± 0.0	6.7 ± 0.3	0.1 ± 0.0	7.1 ± 0.2	0.1 ± 0.0
	8	6.6 ± 0.2	0.0 ± 0.0	6.6 ± 0.3	0.1 ± 0.0	6.6 ± 0.3	0.0 ± 0.0	7.1 ± 0.2	0.1 ± 0.0
	12	6.6 ± 0.2	0.1 ± 0.0	6.6 ± 0.3	0.2 ± 0.0	6.7 ± 0.3	0.0 ± 0.0	7.1 ± 0.2	0.1 ± 0.0

^1^ Values are shown as mean ± standard deviation.

**Table 5:** Muscle strength of each group during the intervention period and their changes from the baseline ^1^.

		Control food		Decanoic Acid Food		Low-Dose Octanoic Acid Food		High-Dose Octanoic Acid Food	
	Week	Measurement Values	Change Values	Measurement Values	Change Values	Measurement values	Change Values	Measurement Values	Change Values
Knee extension strength									
Right, N	0	309.3 ± 21.0		306.4 ± 19.6		254.6 ± 19.9		326.3 ± 30.1	
	4	321.3 ± 17.4	12.0 ± 11.2	333.9 ± 20.5	27.5 ± 14.3	292.9 ± 19.7	38.3 ± 14.6	361.5 ± 26.3	35.3 ± 16.2
	8	363.1 ± 20.3	53.8 ± 13.5 ^†^	400.2 ± 20.7 ^†^	93.8 ± 17.4 ^†^	354.7 ± 21.7 ^†^	100.1 ± 14.1 ^†^	418.1 ± 25.9 ^†^	91.8 ± 18.2 ^†^
	12	356.7 ± 16.5	47.3 ± 14.7^†^	426.0 ± 20.3 ^†^	119.6 ± 17.3 *^,†^	391.3 ± 23.0 ^†^	136.7 ± 19.1 *^,†^	456.8 ± 26.3 *^,†^	130.5 ± 18.3 *^,†^
Left, N	0	291.8 ± 19.9		308.0 ± 21.1		244.0 ± 18.1		323.0 ± 24.3	
	4	315.4 ± 17.4	23.6 ± 11.5	329.2 ± 21.6	21.3 ± 14.3	281.9 ± 17.8	37.9 ± 16.4	345.9 ± 22.9	22.8 ± 11.3
	8	338.3 ± 21.9	46.5 ± 16.4 ^†^	410.1 ± 22.3 ^†^	102.1 ± 15.3 ^†^	357.1 ± 19.2 ^†^	113.1 ± 16.4 *^,†^	413.4 ± 24.4 *^,†^	90.3 ± 18.6 ^†^
	12	356.6 ± 20.1	64.8 ± 15.9 ^†^	433.0 ± 20.2 *^,†^	125.1 ± 13.0 *^,†^	379.6 ± 20.7 ^†^	135.6 ± 21.7 *^,†^	441.6 ± 23.1 *^,†^	118.6 ± 17.9 ^†^
Grip strength									
Right, kg	0	28.6 ± 1.5		27.3 ± 1.6		28.6 ± 1.5		30.0 ± 1.6	
	4	29.4 ± 1.4	0.8 ± 0.5	27.3 ± 1.6	0.1 ± 0.5	28.5 ± 1.5	0.0 ± 0.4	29.8 ± 1.5	−0.2 ± 0.5
	8	29.4 ± 1.3	0.8 ± 0.5	28.2 ± 1.6	1.0 ± 0.7	29.1 ± 1.5	0.5 ± 0.5	30.3 ± 1.5	0.3 ± 0.4
	12	29.3 ± 1.2	0.7 ± 0.6	29.1 ± 1.5	1.9 ± 0.7	29.5 ± 1.6	0.9 ± 0.5	30.8 ± 1.4	0.8 ± 0.6
Left, kg	0	27.4 ± 1.3		26.0 ± 1.5		27.3 ± 1.4		28.3 ± 1.5	
	4	27.6 ± 1.3	0.1 ± 0.4	26.2 ± 1.5	0.2 ± 0.6	27.1 ± 1.4	−0.1 ± 0.4	28.8 ± 1.6	0.5 ± 0.5
	8	27.7 ± 1.3	0.2 ± 0.4	27.3 ± 1.5	1.3 ± 0.6 ^†^	27.6 ± 1.5	0.3 ± 0.4	29.3 ± 1.4	1.0 ± 0.5 ^†^
	12	27.3 ± 1.2	−0.1 ± 0.5	27.7 ± 1.5	1.7 ± 0.7 ^†^	28.0 ± 1.4	0.7 ± 0.4	29.7 ± 1.5	1.4 ± 0.5 ^†^

^1^ Values are shown as mean ± standard deviation. * Significantly different from control group (*p* < 0.05, Dunnett test). ^†^ Significantly different from the baseline (*p* < 0.05, Dunnett test).

**Table 6:** Walking ability of each group during the intervention period and their changes from the baseline ^1^.

		Control Food		Decanoic Acid Food		Low-Dose Octanoic Acid Food		High-Dose Octanoic Acid Food	
	Week	Measurement Values	Change Values	Measurement Values	Change Values	Measurement Values	Change Values	Measurement Values	Change Values
Timed up & go test, s	0	6.7 ± 0.3		7.3 ± 0.3		6.9 ± 0.2		6.7 ± 0.2	
	4	6.4 ± 0.2	−0.4 ± 0.2	6.8 ± 0.2	−0.5 ± 0.3 ^†^	6.7 ± 0.2	−0.2 ± 0.1 ^†^	6.5 ± 0.2	−0.2 ± 0.1
	8	6.2 ± 0.1	−0.6 ± 0.2 ^†^	6.5 ± 0.2 ^†^	−0.9 ± 0.2 ^†^	6.3 ± 0.2	−0.6 ± 0.1 ^†^	6.3 ± 0.2	−0.4 ± 0.1
	12	6.1 ± 0.1 ^†^	−0.6 ± 0.2 ^†^	6.2 ± 0.1 ^†^	−1.2 ± 0.2 ^†^	5.9 ± 0.2 ^†^	−1.0 ± 0.1 ^†^	5.9 ± 0.1 ^†^	−0.8 ± 0.1 ^†^

^1^ Values are shown as mean ± standard deviation. ^†^ Significantly different from the baseline (*p* < 0.05, Dunnett test).

## Data Availability

Data are not available due to commercial restrictions.
